# Preoperative skeletal muscle status is associated with tumor‐infiltrating lymphocytes and prognosis in patients with colorectal cancer

**DOI:** 10.1002/ags3.12570

**Published:** 2022-03-25

**Authors:** Nobuya Daitoku, Yuji Miyamoto, Yukiharu Hiyoshi, Ryuma Tokunaga, Yuki Sakamoto, Hiroshi Sawayama, Takatsugu Ishimoto, Yoshifumi Baba, Naoya Yoshida, Hideo Baba

**Affiliations:** ^1^ Department of Gastroenterological Surgery Graduate School of Medical Sciences Kumamoto University Kumamoto Japan; ^2^ International Research Center for Medical Sciences Kumamoto University Kumamoto Japan

**Keywords:** colorectal cancer, prognosis, sarcopenia, skeletal muscle index, tumor‐infiltrating lymphocytes

## Abstract

**Background:**

Sarcopenia is associated with poor prognosis in patients with colorectal cancer (CRC), but the mechanisms contributing to this association remain unclear. We hypothesized that skeletal muscle status is associated with tumor‐infiltrating lymphocytes (TILs) in patients with CRC. Therefore, this study investigated the clinical effect of sarcopenia and its relationship with the local immune system in CRC patients.

**Methods:**

A total of 256 consecutive patients with CRC who underwent curative resection between 2008 and 2014 were enrolled. Sarcopenia was determined according to the skeletal muscle index (SMI), which was assessed using L3 skeletal muscle mass on axial computed tomography images, and its relationship with patient clinicopathological characteristics and survival was evaluated. Additionally, TILs (CD3^+^, CD8^+^, CD4^+^, and FOXP3^+^ T cells) were assayed by immunohistochemistry. The relationship between TILs and skeletal muscle status was evaluated.

**Results:**

Patients with a lower SMI showed significantly shorter recurrence‐free and overall survival compared with those with a higher SMI. Low expression of TILs was associated with significantly shorter recurrence‐free survival. SMI was significantly correlated with the number of CD3^+^ and CD8^+^ cells in the ordinal logistic regression analysis. Patients with low skeletal muscle status and low CD3^+^ and CD8^+^ cells had an unfavorable prognosis compared with patients with high skeletal muscle status and high CD3^+^ and CD8^+^ cells.

**Conclusion:**

Our data showed an association between skeletal muscle status and local immune cells, and this association may play a pivotal role in the clinical outcome of patients with CRC.

## Graphical abstract

Sarcopenia and TILs.

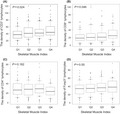

## 1 INTRODUCTION

Colorectal cancer (CRC) is currently the third most common cause of cancer‐related mortality in economically developed countries, and is on track to increase in ranking in the coming decades.^1^ Surgical resection with adjuvant chemotherapy offers the only hope for a cure and can promote long‐term survival in CRC patients. However, CRC recurs in ~30% of patients, and better treatment options are needed to improve the prognosis.^2^, ^3^, ^4^ Moreover, because CRC has a high relapse rate, even after early radical resection, there is a need for additional biomarkers that can complement currently available treatments. This will help to predict early postoperative recurrence and poor prognosis in patients with CRC.

Accumulating evidence suggests that skeletal muscle mass is associated with survival outcomes in patients with various cancers.^5^, ^6^, ^7^ In CRC, several studies have shown that patients with low skeletal muscle mass, as measured by preoperative computed tomography (CT), have poor survival outcomes compared with those with normal skeletal muscle mass.^8^, ^9^ Skeletal muscle measurement using diagnostic CT scans may improve patient management when utilized for prognostic prediction in CRC patients.^10^, ^11^, ^12^ However, the mechanisms underlying the influence of skeletal muscle loss on cancer prognosis remain unclear.

Tumor‐infiltrating lymphocytes (TILs) are an essential histopathological feature of CRC that provides prognostic information. Previous clinical and epidemiological studies found that high levels of TILs were significantly associated with better disease‐specific and overall survival of patients with CRC. In addition, a scoring system called Immunoscore, which summarizes the densities of CD3^+^ and CD8^+^ T‐cell effectors within the tumor and its invasive margins, has been shown to be useful in predicting the clinical outcome of patients with CRC.^11^ High levels of TILs in CRC could also effectively predict response to chemotherapy in patients with CRC.^13^ Interestingly, patients with a higher level of TILs were more likely to benefit from chemotherapy in terms of recurrence risk. As part of the local immune system, tumor infiltration of immune cells could represent host immune reactions against cancer cell proliferation and affect prognosis in patients with CRC. More recently, reports have shown a significant correlation between TILs and sarcopenia. We previously showed that patients with sarcopenia had significantly fewer CD8^+^ cells than patients with extrahepatic cholangiocarcinoma.^14^

Therefore, we hypothesized that skeletal muscle status might be associated with the number of TILs in patients with CRC. A better understanding of the relationship between patient skeletal muscle status and immune cells in the tumor microenvironment may create new opportunities for CRC treatment to target the skeletal muscle. Thus, this study aimed to investigate the role of TILs as a prognostic factor in CRC and the relationship between sarcopenia and the local immune system in CRC patients.

## 2 MATERIALS AND METHODS

### 2.1 Patients

We recruited 256 patients with CRC who had undergone primary tumor resection at Kumamoto University Hospital between April 2008 and December 2014. Patients with histologically verified colorectal adenocarcinoma who had undergone primary tumor resection and enhanced CT at least 1 mo prior to surgery were eligible. Additionally, only patients whose tumors were available for immunostaining of TILs were eligible. Patients who had received preoperative chemotherapy, radiotherapy, or emergent surgery were excluded. Pathological findings were prospectively evaluated according to the seventh edition of the American Joint Committee on Cancer system of CRC. Patients were followed up at 3–6‐mo intervals until death or for at least 5 y. Recurrence‐free survival (RFS) was defined as the time between the surgery date and the date of recurrence or death. Overall survival (OS) was defined as the time between the surgery date and the date of death. All patients provided informed consent, including permission for tumor tissue and the use of images to explore parameters. This study was conducted according to the REporting recommendations for tumor MARKer prognostic studies (REMARK).^15^ The tissue analysis protocol was approved by the Kumamoto University Institutional Review Board of Medical Sciences and conducted at Kumamoto University in accordance with the Declaration of Helsinki and Good Clinical Practice guidelines.

### 2.2 Tissue samples

CRC tissue or paired normal epithelial tissue was obtained at the time of surgical resection, snap‐frozen, and stored at –80°C until use. RNA or cDNA was extracted from frozen sample tissue as described below. Formalin‐fixed paraffin‐embedded tissue samples were used for immunohistochemical staining.

### 2.3 Immunohistochemistry

Paraffin‐embedded tumor sections were dewaxed in xylene and ethanol and autoclaved for 15 min in an antigen retrieval solution to retrieve their antigen epitopes, and endogenous peroxidase activity was blocked with 3% H_2_O_2_. Tissue sections were incubated overnight at 4°C with primary antibodies, including rabbit polyclonal anti‐CD3 (1:200 dilution; ab4055, Abcam, Cambridge, UK) for CD3^+^ T lymphocytes, mouse monoclonal anti‐CD8 (1:300 dilution; clone G10F5, BD Pharmingen, San Diego, CA, USA) for CD8^+^ T lymphocytes, anti‐CD4 (1:300 dilution; clone 10D6, Novocastra, Newcastle, UK) for CD4^+^ T lymphocytes, and anti‐FOXP3 (1:300 dilution; clone 10D6, Novocastra) for FOXP3^+^ T lymphocytes. The secondary antibody was incubated in a ready‐for‐use EnVision–peroxidase system (Dako Japan, Tokyo, Japan). Sections were incubated with horseradish peroxidase‐labeled polymer (EnVision1kit, Dako, Carpinteria, CA, USA) for 30 min at 25°C and incubated with 3,30‐diaminobenzidine tetrahydrochloride (applied as a 0.02% solution containing 0.005% H_2_O_2_ in 0.05 M Tris–HCl; pH 7.6) at 25°C for 5–15 min and counterstained with hematoxylin. We counted each positive lymphocyte in the invasive tumor margin using a BZ‐X700 digital microscope at a magnification of 200× (Keyence) using hybrid cell count software (BZ‐H3C; Keyence; Figure S1).

### 2.4 Measurement and evaluation of skeletal muscle mass

Skeletal muscle area was measured retrospectively using images from CT performed before surgery at the third lumbar vertebra level in the inferior direction, with the patient in the supine position. We briefly measured the number of pixels using a window width of –30 to 150 HU to delineate muscle compartments and compute their cross‐sectional areas in cm^2^ using the Volume Analyzer Synapse Vincent 3D image analysis system (Fujifilm Medical, Tokyo, Japan). The cross‐sectional area of the muscle (cm^2^) at the L3 level computed from each image was normalized by the square of the height (m^2^) to obtain the skeletal muscle index (SMI; cm^2^/m^2^).^16^, ^17^ We divided the patients into quartiles according to the SMI using a sex‐specific categorical variable (Q1 [males: 29.3–43.5, n = 38; females: 25.0–37.3, n = 26], Q2 [males: 43.6–50.3, n = 38; females: 37.3–41.8, n = 27], Q3 [males: 50.5–56.6, n = 38; females: 41.9–45.9, n = 26], and Q4 [males: 57.0–85.0, n = 37; females: 46.0–56.2, n = 26]). We defined the lowest SMI (Q1) as a sarcopenia condition.

### 2.5 Microsatellite instability analysis

Microsatellite instability (MSI) analysis was performed using the *BAT25* and the *BAT26* primer set.^18^ The sense primers were labeled with FAM. Polymerase chain reaction (PCR) was performed, and dye‐labeled PCR products were analyzed with an ABI PRISM 3100 Genetic Analyzer using Genescan 3.7 software (Applied Biosystems, Darmstadt, Germany). A total of 0.5 µL PCR product was mixed with 9.25 µL highly deionized formamide and 0.25 µL DNA Size Standard LIZ 500 (–250; Applied Biosystems). This mixture was denatured for 3 min at 95°C, immediately placed on ice, and separated using an ABI 3130 Genetic Analyzer. Results were analyzed using GeneMapper software (Applied Biosystems). Both tumor and normal tissue samples were analyzed. Samples were divided into two groups: those with one or more of the two markers displaying MSI and those with no instability (microsatellite stable).

### 2.6 Statistical analysis

C ± standard deviation (SD) or median (interquartile range) was calculated according to the data type (parametric or nonparametric), and differences were assessed for significance using Student's *t*‐test or the Mann–Whitney test. Categorical variables were evaluated using the chi‐squared or Fisher's exact test, as appropriate. We conducted ordinal logistic regression analysis to assess associations of the density of TILs with SMI (an ordinal outcome variable [Q1 vs Q2 vs Q3 vs Q4]). Cox proportional hazard regression analyses were performed to identify predictors of prognosis. RFS and OS rates were estimated using the Kaplan–Meier method, and survival curves were compared using the log‐rank test. Statistical significance was set at *P* < .05. All tests were performed using JMP software (v. 13.0.0; SAS Institute, Cary, NC, USA) and R (v. 3.4.4; www.r‐project.org).

## 3 RESULTS

### 3.1 Preoperative SMI and long‐term outcomes

**FIGURE 1:**
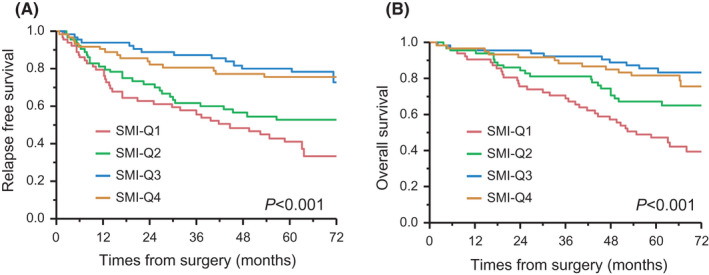
Kaplan–Meier curves for relapse‐free survival and overall survival according to skeletal muscle index (SMI) in patients with colorectal cancer. (A) Relapse‐free survival. (B) Overall survival. SMI was categorized into four quartiles from the lowest (Q1) to the highest (Q4)

We measured the SMI of 256 patients with CRC who underwent primary tumor resection using a preoperative CT scan. Table 1 shows the clinical and pathological features of the 256 cases according to the SMI. The median patient follow‐up was 62.5 mo (95% confidence interval [CI]: 51.4–94.1), as measured by the reverse Kaplan–Meier method. Kaplan–Meier curves were constructed to assess the RFS and OS of CRC patients. In this analysis, patients in the lowest skeletal muscle group (ie, Q1 patients) experienced significantly shorter RFS (5‐y RFS, Q1 vs Q4 = 41.1% vs 75.3%; log‐rank *P* < .001) and OS (5‐y OS, Q1 vs Q4 = 47.1% vs 81.8%; log‐rank *P* < .001) compared with those in the highest skeletal muscle group (Figure 1).

**TABLE 1:** Patient characteristics

Variables	Skeletal Muscle Index					*P* value
	All	Q1	Q2	Q3	Q4	
	n (%)	n (%)	n (%)	n (%)	n (%)	
All	256	64	65	65	63	
Age, y (range)	69 (19–93)	70 (44–88)	71 (19–90)	69 (25–90)	68 (33–93)	.12
Gender						
Male	151 (59)	38 (59)	38 (59)	38 (59)	37 (59)	1.00
Female	105 (41)	26 (41)	27 (42)	26 (41)	26 (41)	
Body mass index, kg/m^2^ (range)	22.0 (13.7–41.1)	20.0 (13.7–41.1)	22.0 (15.7–31.3)	21.8 (15.9–35.4)	24.4 (16.4–32.4)	<.001
Tumor location						
Right‐sided	75 (29)	23 (36)	16 (25)	22 (34)	14 (22)	.28
Left‐sided	89 (35)	22 (34)	24 (37)	16 (25)	27 (43)	
Rectum	92 (36)	19 (30)	25 (38)	26 (41)	22 (35)	
Tumor depth						
pT1	24 (9)	3 (95)	5 (8)	8 (13)	8 (13)	.33
pT2‐4	232 (91)	61 (5)	60 (92)	56 (88)	55 (87)	
Lymph node metastases						
Present	104 (41)	29 (45)	27 (42)	27 (42)	21 (33)	.56
Absent	152 (59)	35 (55)	38 (58)	37 (58)	42 (67)	
Disease stage (AJCC)						
I	68 (27)	14 (22)	15 (23)	20 (31)	19 (30)	.028
II	72 (28)	17 (27)	17 (26)	17 (27)	21 (33)	
III	68 (27)	17 (27)	13 (20)	23 (36)	15 (24)	
IV	48 (19)	16 (25)	20 (30)	4 (6)	8 (13)	
Tumor differentiation						
Well to moderate	221 (86)	56 (88)	57 (88)	52 (81)	56 (89)	.59
Poor	35 (14)	8 (12)	8 (12)	12 (19)	7 (11)	
Lymphatic invasion						
Present	107 (42)	28 (44)	28 (43)	28 (44)	23 (37)	.80
Absent	149 (58)	36 (56)	37 (57)	36 (56)	40 (63)	
Venous invasion						
Present	137 (54)	34 (53)	37 (57)	30 (47)	36 (57)	.62
Absent	119 (46)	30 (47)	28 (43)	34 (53)	27 (43)	
MSI status						
MSS/MSI‐Low	226 (88)	58 (91)	58 (89)	54 (84)	56 (88.9)	.99
MSI‐High	30 (12)	6 (9)	27 (41)	26 (40.6)	26 (41.3)	

Note:

Q1 [male: SMI 29.3–43.5; female: SMI 25.0–37.3], Q2 [male: SMI 43.6–50.3; female: SMI 37.3–41.8], Q3 [male: SMI 50.5–56.6; female: SMI 41.9–45.9], and Q4 [male: SMI 57.0–85.0; female: SMI 46.0‐ 56.2].

Abbreviations: CA19‐9, carbohydrate antigen 19‐9; CEA, carcinoembryonic antigen; MSI, microsatellite instability; MSS, microsatellite‐stable; SMI, skeletal muscle index.

### 3.2 Preoperative intratumoral TILs and recurrence‐free survival

**FIGURE 2:**
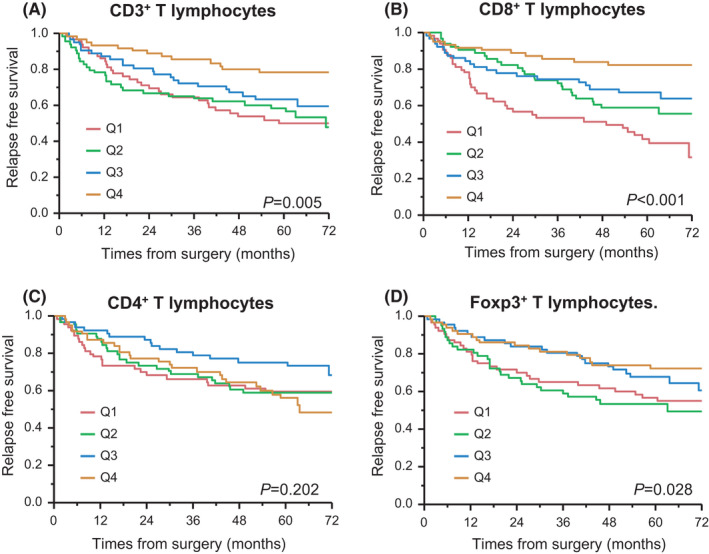
Kaplan–Meier curves for recurrence‐free survival according to quartiles (Q1–Q4) of TILs in patients with colorectal cancer. The density of tumor‐infiltrating lymphocytes was categorized into four quartiles from the lowest (Q1) to the highest (Q4). (A) CD3^+^ T lymphocytes. (B) CD8^+^ T lymphocytes. (C) CD4+ T lymphocytes. (D) FOXP3^+^ T lymphocytes

Kaplan–Meier curves were constructed to assess the RFS of CRC patients according to intratumoral TILs. We regarded CD3^+^, CD8^+^, CD4^+^, and FOXP3^+^ T lymphocytes as local immune status. The local immune cells were evaluated by immunohistochemistry (Figure S1) and are presented in Figure 2, which shows the Kaplan–Meier curves for survival in CRC patients according to TILs. There were recurrences in 47 cases in this study. The recurrence types of CRC were 24 (51.1%) liver metastases, 13 (27.7%) lung metastases, six (12.8%) lymph node metastases, four (8.5%) local recurrences, four (8.5%) peritoneal disseminations, and one (2.1%) ovary (Table 1). There were significant differences between CD8^+^ lymphocytes and lung recurrence and lymph node recurrence (lung, *P* = .045; lymph node, *P* = .050; Table S1). CD4^+^ and FOXP3^+^ lymphocytes were significantly lower in patients with lymph node recurrence (CD4, *P* = .039; FOXP3, *P* = .027; Table S1). Patients with low expression of TILs showed a significantly shorter RFS except CD4^+^ TILs (CD3^+^, *P* = .005; CD8^+^, *P* < .001; CD4^+^, *P* = .202; FOXP3^+^, *P* = .028).

### 3.3 Relationship between skeletal muscle status and density of TILs

**FIGURE 3:**
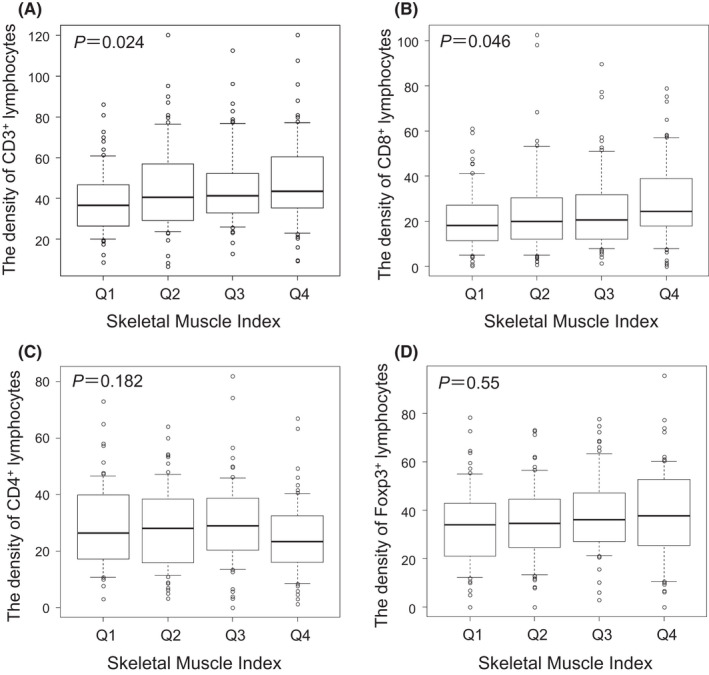
Correlation between intratumoral TILs and skeletal muscle index in patients with CRC. (A) CD3^+^ T lymphocytes. (B) CD8^+^ T lymphocytes. (C) CD4^+^ T lymphocytes. (D) FOXP3^+^ T lymphocytes

To investigate the impact of skeletal muscle status on local immune systems and cancer progression, we analyzed the relationship between TILs and SMI. In addition, we evaluated the association with clinicopathological factors, such as albumin and CRP (C‐reactive protein). It was inferred that they would be related to sarcopenia. In our analysis, SMI was significantly correlated with the number of CD3^+^ and CD8^+^ cells, as shown in Figure 3. Ordinal logistic regression analysis showed that CD3^+^ and CD8^+^ cells were independently associated with the SMI in patients with CRC, like other factors such as albumin and CRP (Table 2).

**TABLE 2:** Ordinal logistic regression analysis to assess independent associations of tumor‐infiltrating lymphocytes with the skeletal muscle index

	Univariate analysis		Multivariate analysis^a^	
	OR (95% CI)	*P* value	OR (95% CI)	*P* value
Albumin (g/dl)	6.35 (1.64–2.50)	.**001**	4.91 (1.45–2.53)	.**001**
CRP (mg/dl)	1.73 (1.02–1.28)	.**019**	1.61 (1.17–1.28)	.**026**
WBC(/μl)	0.30 (0.60–1.22)	.496	0.18 (0.72–1.13)	.662
Neutrophil (%)	2.43 (1.09–1.49)	.**004**	1.51 (1.21–1.43)	.**031**
Monocyte (%)	0.82 (0.72–1.19)	.152	0.52 (0.51–1.71)	.304
Lymphocyte (%)	2.89 (1.60–2.14)	.**001**	1.97 (1.55–1.72)	.**011**
NLR	2.71 (1.53–2.48)	.**002**	1.95 (1.28–2.27)	.**011**
Vascular emboli	0.02 (0.77–1.21)	.952	0.99 (0.55–1.42)	.103
Lymphatic invasion	0.35 (0.86–1.31)	.451	0.17 (0.47–1.34)	.678
CD3 (for 10 increase)	1.17 (1.05–1.31)	.**005**	1.18 (1.05–1.32)	.**006**
CD8 (for 10 increase)	1.17 (1.04–1.33)	.**011**	1.16 (1.01–1.33)	.**037**
CD4 (for 10 increase)	0.90 (0.78–1.05)	.186	0.87 (0.74–1.01)	.075
Foxp3 (for 10 increase)	1.11 (0.98–1.27)	.111	1.08 (0.94–1.24)	.263

Abbreviations: CEA, carcinoembryonic antigen; CI, confidence interval; CRP, C reactive protein; MSI, microsatellite instability; MSI‐H, microsatellite instability‐high; MSS, microsatellite‐stable; NLR, neutrophil/lymphocyte ratio; OR, odds ratio; WBC, white blood cell.

^a^ Multivariate ordinal logistic regression analysis model included age (continuous), sex (male vs female), location (right vs left vs rectum), differentiation (well, moderate vs poor), Stage (I vs II vs III vs IV), MSI (MSS vs MSI‐H), CEA (high vs normal).

Bold type indicates values significant at *P* < .05.

### 3.4 Prognostic value of skeletal muscle status and TILs in patients with CRC

**FIGURE 4:**
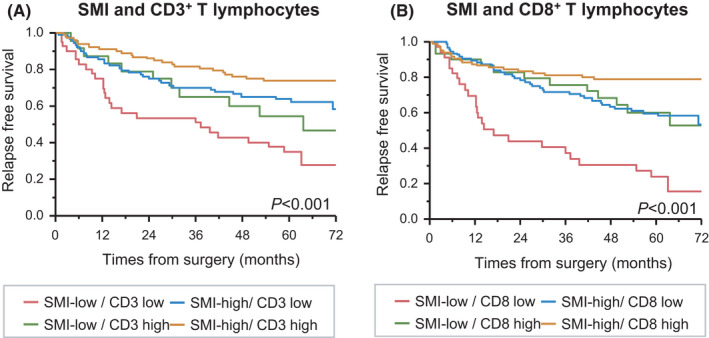
Kaplan–Meier curves for relapse‐free survival according to skeletal muscle index and tumor infiltrating lymphocytes. (A) SMI and CD3^+^ T lymphocytes. B) SMI and CD8^+^ T lymphocytes

Next, we evaluated the prognostic value of the combination of skeletal muscle status and TILs. The patients were divided into four groups according to SMI (SMI‐low Q1 vs SMI‐high Q2–4) and TIL status (high vs low by median). Patients with SMI‐low and CD3‐low had a significantly shorter RFS than the other patients (*P* < .001; Figure 4A). Patients with SMI‐low and CD8‐high had a favorable prognosis compared with the other patients (*P* < .001; Figure 4B).

The multivariate Cox regression analyses showed the combination of SMI‐low and low‐CD3 or CD8 were independent predictors of RFS in CRC patients (SMI/CD3 TILs: hazard ratio [HR] = 2.90, 95% CI = 1.82–4.62, *P* < .001; SMI/CD8 TILs: HR = 3.01, 95%CI = 1.84–4.90, *P* < .001; Table 3).

**TABLE 3:** Univariate and multivariate Cox regression analysis for relapse‐free survival in CRC patients

	Univariate analysis		Multivariate analysis^a^	
	HR (95% CI)	*P* value	HR (95% CI)	*P* value
Albumin (albumin‐low vs albumin‐high)	2.19 (1.47–3.26)	**<.001**	1.92 (1.25–2.97)	.**003**
CRP (CRP‐high vs CRP‐low)	1.51 (1.02–2.26)	.**044**	1.39 (0.92–2.14)	.123
WBC (WBC‐high vs WBC‐low)	1.02 (0.69–1.51)	.921		
Neutrophil (neutrophil‐high vs neutrophil‐low)	1.62 (1.09–2.43)	.**018**	1.23 (0.82–1.87)	.324
Monocyte (monocyte‐high vs monocyte‐low)	1.42 (0.95–2.12)	.085		
Lymphocyte (lymphocyte‐low vs lymphocyte‐high)	1.68 (1.13–2.52)	.**011**	1.26 (0.84–1.92)	.268
NLR (NLR‐high vs NLR‐low)	1.77 (1.19–2.67)	.**005**	1.30 (0.86–1.99)	.211
Vascular emboli (negative vs positive)	2.25 (1.51–3.35)	**<.001**	1.02 (0.64–1.63)	.947
Lymphatic invasion (negative vs positive)	2.03 (1.35–3.11)	**<.001**	1.05 (0.53–1.79)	.867
SMI (SMI‐low vs SMI high)	2.46 (1.64–3.70)	**<.001**	2.35 (1.53–3.59)	**<.001**
CD3 positive TILs (CD3‐low vs CD3‐high)	1.85 (1.23–2.80)	.**003**	1.89 (1.24–2.87)	.**003**
CD8 positive TILs (CD8‐low vs CD8‐high)	2.51 (1.64–3.83)	**<.001**	1.86 (1.19–2.90)	.**006**
SMI / CD3 TILs (SMI‐low / CD3‐low vs others)	2.83 (1.81–4.43)	**<.001**	2.90 (1.82–4.62)	**<.001**
SMI / CD8 TILs (SMI‐low / CD8‐low vs others)	3.93 (2.49–6.19)	**<.001**	3.01 (1.84–4.90)	**<.001**

Abbreviations: CI, confidence interval; CRP, C reactive protein; HR, hazard ratio; NLR, neutrophil/lymphocyte ratio; SMI, skeletal muscle index; TILs, tumor‐infiltrating lymphocytes; WBC, white blood cell.

^a^ Multivariate cox regression analysis initially included age (continuous), sex (male vs female), location (right vs left vs rectum), differentiation (well, moderate vs poor), Stage (I vs II vs III vs IV), MSI (MSS vs MSI‐H), and CEA (high vs normal). A stepwise Akaike's Information Criterion (AIC) method was used to select variables in the final models, and age, differentiation, Stage, and CEA variables remained in the final model.

Bold type indicates values significant at *P* < .05.

## 4 DISCUSSION

This study comprehensively analyzed the relationship between skeletal muscle status and TILs in patients with CRC who underwent curative resection. Cases with a lower SMI and low expression of TILs showed significantly shorter RFS and OS compared with cases with a higher SMI. SMI was significantly correlated with the number of CD3^+^ and CD8^+^ cells in the ordinal logistic regression analysis. To the best of our knowledge, this is the first report showing that CD3^+^ and CD8^+^ lymphocytes correlated with skeletal muscle status and prognosis in patients with CRC. Moreover, patients with low skeletal muscle status and low CD8^+^ cells had an unfavorable prognosis compared with patients with high skeletal muscle status and high CD8^+^ cells.

Although sarcopenia has been associated with morbidity and mortality in previous reports, the mechanisms are not completely understood. Some reports have reported that skeletal muscle and adipose tissues secrete several different cytokines and peptides.^19^, ^20^ Thus, these peptides may affect the immune system, especially natural killer cells and innate immune cells, which help to control intracellular infectious agents and cancers. However, there have been few reports showing this relationship in clinical samples. We comprehensively analyzed systemic immune cells from clinical data and assayed TILs using immunohistochemistry.^21^ There is growing evidence that the local immune response plays an important role in the progression of a variety of solid tumors.^10^, ^11^, ^14^, ^22^ In previous studies, evidence has suggested that TILs may be correlated with improved clinical outcomes in patients with CRC. The prognostic impact of the tumor microenvironment has been clearly established for precancerous lesions, primary tumors, and metastasis.^13^, ^23^, ^24^ Consistent with previous reports, this study confirmed the usefulness of CD3^+^ and CD8^+^ T‐cell densities as prognostic factors.^11^, ^23^ Importantly, in localized CRC, TILs were found to be more efficient at stratifying patient prognosis and predicting the benefits of adjuvant cytotoxic chemotherapy than TNM staging.^11^, ^13^ In particular, TILs, including CD8^+^ lymphocytes, have been associated with favorable clinical outcomes in multiple tumor types. The present study also revealed that the CD8^+^ lymphocyte count in CRC tissues was associated with patient prognosis. In this study, there were recurrences in 47 cases. Patients with liver recurrence tended to show fewer CD8^+^ lymphocytes (*P* = .077; Table S1). Moreover, CD8^+^ lymphocytes were significantly lower in patients with lung and lymph node recurrences (lung, *P* = .045; lymph node, *P* = .050; Table S1). Thus, some TILs might be associated with recurrence. However, the relationship between TILs and recurrence types is unclear because of the small number of cases.

We also showed that sarcopenia was significantly correlated with the number of CD3^+^ and CD8^+^ cells. Previously, Kitano et al showed that sarcopenia and systemic or local immune cells may interact with each other and play a pivotal role in clinical outcomes for patients with extrahepatic cholangiocarcinoma.^14^ Our results do not demonstrate whether skeletal muscle loss was the cause or the result of local immunity suppression. In vitro experiments are needed to show this mechanism. However, Chatraw et al^21^ reported that protein energy malnutrition (PEM) led to lower numbers of CD8^+^ lymphocytes and increased the incidence and severity of infection in mice. As a result, PEM‐induced muscle loss might decrease immune activity. We support this mechanism and believe that skeletal muscle loss is the cause of suppression of local immunity.^21^ Furthermore, Rundqvist et al reported CD8^+^ T cells are metabolically enhanced by exercise in mice.^25^ This exercise‐induced reduction in tumor growth was dependent on CD8^+^ T cells. Both of these reports were from mouse models, but they support our mechanism. Therefore, in patients with malnutrition, decreased antigen‐presenting ability, and decreased interleukin (IL)‐2 and interferon (IFN)‐γ production may be observed, leading to decreased activation and migration CD8^+^ lymphocytes. Moreover, SMI was significantly correlated with the number of CD3^+^ and CD8^+^ cells in our study. Lutz and Quinn revealed that IL‐15 was highly expressed in skeletal muscle tissue and declined in aging rodent models, and that IL‐15 was required to develop and maintain natural killer lymphocytes.^26^ They suggested that decreased IL‐15 levels during aging constitute a common mechanism for sarcopenia and immune senescence. Another study reported that immune senescence resulted from an imbalance in inflammatory and antiinflammatory mechanisms, and cancer, as well as aging, was one such condition.^27^ In such inflammatory conditions, the proinflammatory cytokine IL‐6 is elevated, leading to one of the essential components of sarcopenia.

In this study, patients with low skeletal muscle status and low CD8^+^ cells showed an unfavorable prognosis compared with patients with high CD8^+^ cells. This result shows that low expression of TILs may contribute to poor prognosis in patients with CRC. Accumulating evidence demonstrates a significant role of tumor microenvironment cells in both cancer progression and tumor‐induced muscle loss via the production of multiple inflammatory factors.^28^ However, the mechanisms of intratumoral CD8^+^ cells in sarcopenia, and how they might affect prognosis, remain unclear. Our data may provide new ideas for the exploration of potential mechanisms.

Recently, several studies showed a relationship between systemic inflammation and local immune systems, such as TILs or tumor‐associated macrophages, in patients with gastrointestinal cancers.^24^, ^29^ Okadome et al hypothesized that there was a relationship between nutritional status and local immune competence with Prognostic Nutritional Index (PNI) and immunobiological features in patients with esophageal cancer.^22^ Associations between systemic and local immune responses should be analyzed to better understand the tumor microenvironment in CRC patients.

This study had some limitations. First, our dataset was obtained from a single institution and was collected retrospectively. Second, our study only included preoperative SMI status and did not consider the postoperative host status for prognosis. Fourth, there were significant differences between SMI and pathological stage (Table 1, *P* = .028). These differences might affect the present study results. Actually, these differences might affect the prognosis of CRC patients according to SMI. However, we included several clinical and pathological factors in ordinal logistic regression analysis and multivariate Cox regression analysis. Therefore, we thought these influences were minimal. In this study, although we revealed that sarcopenia is associated with the immune system in CRC patients, it remains unclear whether sarcopenia is a cause or result of the immune system findings, or whether another confounding factor exists. Therefore, further analyses, such as in vivo analyses, are required. Nevertheless, this was the first study to investigate the relationship between SMI and TILs in patients undergoing curative surgery for CRC.

In conclusion, the relationship between skeletal muscle status and TILs may play a pivotal role in the clinical outcomes of patients with CRC who have undergone curative resection. These mechanisms may be exploited in cancer therapeutics, such as immune therapy or preoperative nutritional intervention. Future studies are needed to confirm our findings and examine other potential mechanisms by which skeletal muscle status affects TILs.

## DISCLOSURE

Funding: No funding source.

Conflict of Interest: Author Hideo Baba is an editorial board member of Annals of Gastroenterological Surgery: The funding source had no role in the design, practice, or analysis of this study.

Presentation: We have not submitted this article elsewhere.

Ethical Approval: Institutional Review Board of Kumamoto University (number 1047).

Informed Consent: Consent was obtained from the patients and families according to Institutional Review Board protocols.

## Supporting information

Fig S1

Table S1
