# Supplementary material for: Preoperative skeletal muscle status is associated with tumor‐infiltrating lymphocytes and prognosis in patients with colorectal cancer
Source: Ann Gastroenterol Surg. 2022 Mar 25;6(5):658–66. doi: 10.1002/ags3.12570 (PMC9444852; doi:10.1002/ags3.12570)
Supplement: Supplementary file 1 — Fig S1 [file AGS3-6-658-s002.docx]

**Supplemental Figure 1. Immunohistochemical staining of TILs in colorectal cancer.**

(a) Representative images of TILs expression (CD3, CD8, CD4, Foxp3) at ×40 magnification. (b) cell counting by hybrid cell count software. TILs-positive cells are shown as red dots.
