# Supplementary material for: Preoperative skeletal muscle status is associated with tumor‐infiltrating lymphocytes and prognosis in patients with colorectal cancer
Source: Ann Gastroenterol Surg. 2022 Mar 25;6(5):658–66. doi: 10.1002/ags3.12570 (PMC9444852; doi:10.1002/ags3.12570)
Supplement: Supplementary file 2 — Table S1 [file AGS3-6-658-s001.docx]

**Supplementary table 1: Correlation between tumor infiltrating lymphocytes, skeletal muscle index and recurrence types in patients with colorectal cancer.**

|  | | | Tumor infiltrating lymphocytes | | | | | | | | | | |
| --- | --- | --- | --- | --- | --- | --- | --- | --- | --- | --- | --- | --- | --- |
|  |  |  | SMI | | | CD3 | | CD8 | | CD4 | | Foxp3 | |
| Recurrence types | n |  | SMI-Low | SMI-High | *P* value | cells/field | *P* value | cells/field | *P* value | cells/field | *P* value | cells/field | *P* value |
| Liver |  |  |  |  | 0.210 |  | 0.142 |  | 0.077 |  | 0.624 |  | 0.700 |
| present | 24 |  | 15 | 9 |  | 107.4 |  | 103.0 |  | 135.6 |  | 122.9 |  |
| absent | 232 |  | 114 | 118 |  | 130.7 |  | 131.1 |  | 127.8 |  | 129.0 |  |
| Lung |  |  |  |  | 0.158 |  | 0.128 |  | **0.045** |  | 0.914 |  | 0.147 |
| present | 13 |  | 9 | 4 |  | 98.1 |  | 88.4 |  | 126.3 |  | 99.5 |  |
| absent | 243 |  | 120 | 123 |  | 130.1 |  | 130.6 |  | 128.6 |  | 130.1 |  |
| Lymph node |  |  |  |  | 0.415 |  | 0.065 |  | **0.050** |  | **0.039** |  | **0.027** |
| present | 6 |  | 4 | 2 |  | 73.4 |  | 69.9 |  | 66.9 |  | 62.3 |  |
| absent | 250 |  | 125 | 125 |  | 129.8 |  | 129.9 |  | 130.0 |  | 130.1 |  |
| Local recurrence |  |  |  |  | 0.310 |  | 0.681 |  | 0.151 |  | 0.691 |  | 0.775 |
| present | 4 |  | 3 | 1 |  | 113.3 |  | 75.8 |  | 143.1 |  | 118.0 |  |
| absent | 252 |  | 126 | 126 |  | 128.7 |  | 129.3 |  | 128.3 |  | 128.7 |  |
| Peritoneal dissemination |  |  |  |  | 0.987 |  | 0.529 |  | 0.346 |  | 0.948 |  | 0.520 |
| present | 4 |  | 2 | 2 |  | 151.6 |  | 163.1 |  | 130.9 |  | 104.9 |  |
| absent | 252 |  | 127 | 125 |  | 128.1 |  | 128.0 |  | 128.5 |  | 128.9 |  |
| Ovary |  |  |  |  | 0.236 |  | 0.115 |  | 0.320 |  | 0.612 |  | 0.834 |
| present | 1 |  | 0 | 1 |  | 12.0 |  | 55.0 |  | 91.0 |  | 113.0 |  |
| absent | 255 |  | 129 | 126 |  | 129.0 |  | 128.9 |  | 128.6 |  | 128.6 |  |

SMI Low defined (Q1-Q2), SMI High defined (Q3-Q4), SMI; Q1 [male: SMI 29.3–43.5; female: SMI 25.0–37.3], Q2 [male: SMI 43.6–50.3; female: SMI 37.3–41.8], Q3 [male: SMI 50.5–56.6; female: SMI 41.9–45.9], and Q4 [male: SMI 57.0–85.0; female: SMI 46.0– 56.2], Tumor infiltrating lymphocytes (CD3, CD8, CD4, Foxp3) were evaluated as a continuous variable for its relationship with the recurrence.
